# The effect of executive walk rounds on nurse safety climate attitudes: A randomized trial of clinical units

**DOI:** 10.1186/1472-6963-5-28

**Published:** 2005-04-11

**Authors:** Eric J Thomas, J Bryan Sexton, Torsten B Neilands, Allan Frankel, Robert L Helmreich

**Affiliations:** 1 Department of Internal Medicine, The University of Texas Medical School at Houston, Houston, TX, USA; 2 Department of Anesthesiology and Critical Care Medicine, Johns Hopkins Quality and Safety Research Group, The Johns Hopkins University School of Medicine, Baltimore, USA; 3 Center for AIDS Prevention Studies (CAPS), University of California, San Francisco, San Francisco, CA, USA; 4 Partners Healthcare System, Prudential Tower, Boston, MA, USA; 5 Department of Psychology, The University of Texas at Austin, Austin, TX, USA

## Abstract

**Background:**

Executive walk rounds (EWRs) are a widely used but unstudied activity designed to improve safety culture in hospitals. Therefore, we measured the impact of EWRs on one important part of safety culture – provider attitudes about the safety climate in the institution.

**Methods:**

Randomized study of EWRs for 23 clinical units in a tertiary care teaching hospital. All providers except physicians participated. EWRs were conducted at each unit by one of six hospital executives once every four weeks for three visits. Providers were asked about their concerns regarding patient safety and what could be done to improve patient safety. Suggestions were tabulated and when possible, changes were made. Provider attitudes about safety climate measured by the Safety Climate Survey before and after EWRs. We report mean scores, percent positive scores (percentage of providers who responded four or higher on a five point scale (agree slightly or agree strongly), and the odds of EWR participants agreeing with individual survey items when compared to non-participants.

**Results:**

Before EWRs the mean safety climate scores for nurses were similar in the control units and EWR units (78.97 and 76.78, P = 0.458) as were percent positive scores (64.6% positive and 61.1% positive). After EWRs the mean safety climate scores were not significantly different for all providers nor for nurses in the control units and EWR units (77.93 and 78.33, P = 0.854) and (56.5% positive and 62.7% positive). However, when analyzed by exposure to EWRs, nurses in the control group who did not participate in EWRs (n = 198) had lower safety climate scores than nurses in the intervention group who did participate in an EWR session (n = 85) (74.88 versus 81.01, P = 0.02; 52.5% positive versus 72.9% positive). Compared to nurses who did not participate, nurses in the experimental group who reported participating in EWRs also responded more favorably to a majority of items on the survey.

**Conclusion:**

EWRs have a positive effect on the safety climate attitudes of nurses who participate in the walk rounds sessions. EWRs are a promising tool to improve safety climate and the broader construct of safety culture.

## Background

Many hospitals are implementing executive walk rounds (EWRs), a widely used but unstudied activity to improve patient safety [1,2]. EWRs vary from hospital to hospital, but in general they consist of visits by hospital executives to patient care areas to discuss patient safety issues with providers. EWRs enlist leadership to break down the significant barriers to discuss human error in healthcare. The executive may ask providers to discuss specific events or general processes that could put patients at risk for harm, they ask for suggestions to improve safety, and verbalize their commitment to improving safety. Discussions are documented and lead to action which is followed by feedback to participants. EWRs help hospitals identify opportunities to improve care processes by utilizing the wisdom of frontline providers, they demonstrate the executives' and the organization's commitment to patient safety, and they may improve provider attitudes about safety-related issues. These attitudes are an important part of what is often called a hospital's safety culture [3].

Improving safety culture is The National Quality Forum's first of thirty safe practices for better healthcare [4]. The concept is derived from industries and organizations such as aerospace (NASA), nuclear power, aviation, and the military (naval aircraft carriers and nuclear submarines) that are known for their ability to reliably deal with risky processes [3]. One important driver of safety in these settings is a very explicit commitment to safety by leaders. Other components of a good safety culture include a focus on system improvement instead of blaming individuals for mistakes, reporting and learning from errors, and infrequent unsafe acts. A defective safety culture was highlighted as one of the organizational causes of the recent space shuttle Columbia accident [5] and has been cited as causes of high profile adverse events in hospitals.

Hospitals are feeling pressure to act to improve the safety-related attitudes that are part of safety culture [4]. The true prevalence of EWRs is not known, but at least 200 hospitals have used EWRs through participation in Institute for Healthcare Improvement collaboratives, and nine hospitals in Boston are participating in a 3 year study of EWRs. Over 100 more have requested a database to collect EWR data. However, despite their face validity in the eyes of these early adopters, no evaluation of EWRs has been reported so many questions exist about how EWRs should be conducted and whether or not they are effective. The purpose of this study was to measure the impact of EWRs on one important driver of safety culture – provider attitudes about safety climate as measured by The University of Texas Safety Climate Survey.

## Methods

### Overview

Safety *culture *has been be defined as "the product of individual and group values, attitudes, perceptions, competencies, and patterns of behavior that determine the commitment to, and the style and proficiency of, an organization's health and safety management [3]." Safety *climate*, the primary outcome measure in this study, is comprised of the provider attitudes about patient safety. Safety climate is a part of safety culture (attitudes are part of both definitions) and we measure it with the Safety Climate Survey (described in detail below). We theorized that EWRs would improve individual provider attitudes, which in turn would lead to improved safety climate (the composite of provider attitudes).

Our primary hypothesis was that EWRs would improve safety climate in clinical units. An underlying assumption was that attitudes would improve even among individual providers who did not directly participate in EWRs, due to a spill-over effect from the providers who participated in EWRs to those who did not. The existing model for EWRs has not tried to target every provider in a clinical area. We hypothesized that we would not have to expose all providers in a clinical unit to EWRs in order to improve the overall unit climate.

This was the first detailed study of EWRs so we had several secondary (but a-priori) exploratory hypotheses: 1) that the effect of EWRs would be stronger for providers who participated in EWRs; 2) that the effect of EWRs would vary by provider type; and 3) that some individual items on the Safety Climate Survey would be more responsive to EWRs than others. To test these hypotheses, we randomized units in a hospital to receive EWRs or usual care (the control group). The Safety Climate Survey was administered prior to, and after EWRs.

### Participants and setting

The study was conducted at Memorial Hermann Hospital, a 711 bed urban tertiary care teaching hospital that contained adult and children's hospitals. We grouped into "units" the inpatient clinical areas (such as individual ICUs or wards) that cared for similar types of patients or provided similar services. The departments of radiology, pharmacy, respiratory therapy, and physical therapy/occupational therapy were each considered a unit and not grouped with clinical areas. The Emergency Department was not included. After this grouping process there were 23 units (the clinical care areas plus respiratory therapy, radiology, pharmacy, physical therapy/occupational therapy) eligible for randomization. A random number generator was used to allocate units to receive the executive walk round intervention or to be in the control group.

### Intervention

Six executives (2 Vice Presidents and 4 Assistant Vice Presidents) participated in the intervention. Our rationale for choosing executives was to have executives visit units that they also supervised. There was no literature to inform the frequency of visits so the executives and investigators decided that the executive should visit their units during the daytime approximately once every four weeks for a total of 3 visits. Each executive was accompanied by the hospital's Patient Safety Officer and a staff member from the Performance Improvement Department. EWRs were scheduled in advance with managers for each unit. The executive met with providers either in a common work area on the unit such as the nurses station, or in a conference room. All providers present at that time were encouraged to attend (some providers had to continue patient care), but attendance was not mandatory. The executives had met with the Patient Safety Officer before the intervention began to review patient safety concepts, to educate the executives about EWRs, and to review the questions to ask during EWRs. These questions (Table 1) were generated from the literature [1] and the hospital's patient safety committee. Before asking the questions the executives stated that patient safety was a priority for the hospital, that the purpose of their visit was to foster a culture that encourages open communication and identifies ways to improve systems, that all comments would be kept confidential, and that no individual would be held accountable for system flaws, for revealing errors, or voicing their concerns. We did not enforce one standard script for all executives. Walk round sessions lasted 30–60 minutes. At the close of each session the providers were asked to tell two other providers in their unit who did not attend EWRs about the session. This was done to try and magnify the effect of the intervention and improve attitudes even among those who did not directly participate in an EWR.

**Table 1:** Examples of questions asked by executives during walk rounds.

Have there been any "near misses" that almost caused patient harm but didn't?
Have we harmed any patients recently?
What aspects of the environment are likely to lead to the next patient harm?
Is there anything we could do to prevent the next adverse event?
Can you think of any events in the past days that have resulted in prolonged hospitalization for a patient?
Can you think of a way in which the system or your environment fails you on a consistent basis?
What specific intervention from leadership would make the work you do safer for patients?
What would make this executive walk rounds more effective?

### Outcome measure

The primary outcome measure was safety climate, measured using the Safety Climate Survey. The Safety Climate Survey is derived from similar surveys in commercial aviation [6] that measured safety-related attitudes of cockpit crew members. We adapted the aviation survey for healthcare by incorporating existing concepts from healthcare (especially research on safety [7] and organizational culture [8]), conducting focus groups with health care providers, consulting subject matter experts, and field testing items. The first healthcare version was for intensive care providers [9-11]. Our factor analysis of that survey identified a 7 item scale that we called the Safety Climate Scale. We then added items to the questionnaire which have been linked to safety and performance outcomes in prior aviation research [12,13], as well items that were identified through discussions with hospital executives, quality experts, and other end-users. This resulted in the 21 item Safety Climate Survey that uses a 5-point likert scale where 1 = disagree strongly and 5 = agree strongly. Excluding this study, over 8,000 healthcare providers in 251 clinical areas in 52 hospitals have completed the survey and the survey has been endorsed by the Institute for Healthcare Improvement.

We administered the baseline Safety Climate Survey between September 1^st ^and October 15, 2002. EWRs occurred between late October 2002 and January 31, 2003. The survey was re-administered during March and April 2003. We surveyed Registered Nurses, Licensed Vocational Nurses, Nurse Managers, Pharmacists, technicians, Respiratory Therapists, Physical Therapists, Occupational Therapists, Speech Therapists, and Dieticians. Float pool staff were excluded although some non-float pool providers also worked in more than one unit. We did not survey physicians because many of them did not spend enough time on a specific unit to notice changes in safety climate over a short period of time, and their schedules made it likely that they would not be exposed to the intervention. Human Resources generated lists of eligible providers and unit managers reviewed the lists for accuracy. Managers distributed the baseline surveys at meetings or by placing them in mail boxes. To improve response rates they redistributed surveys every two weeks during each administration period. The post-EWRs surveys were administered as described for the baseline surveys and some providers completed the survey as part of previously scheduled in-service training. We surveyed day and evening shifts (even though walk rounds only occurred during the day) to measure if there was a spill-over effect that could influence the overall unit safety climate.

The study was approved by the University of Texas Health Science Center at Houston Committee for the Protection of Human Subjects.

### Statistical analysis

We could find no research that used EWRs as an intervention to help us calculate sample size a priori so we used the entire hospital (except the emergency department) divided into 23 units as described above. We transformed the five point response scale on the safety climate survey to a 100 point scale and calculated means. The 100 point scale is better understood by hospital administrators and providers. We also calculated the percent positive safety climate score: the percent of respondents in a clinical unit who responded 4 or 5 (agree slightly or agree strongly) on the five point scale.

Our primary hypothesis was that units randomized to receive EWRs would have higher mean safety climate scores than control units. A secondary, a-priori, hypothesis was that some individual items on the SCS would be more responsive to EWRs than others. The five items were: 1) This institution is doing more for patient safety now, than it did one year ago (item 15); 2) The senior leaders in my hospital listen to me and care about my concerns (item 3); 3) Patient safety is constantly reinforced as the priority in this clinical area (item 19); 4) Leadership is driving us to be a safety-centered institution (item 5); and 5) I would feel safe being treated here as a patient (item 11).

Another a-priori hypothesis was that the effect of EWRs would vary by provider type and that the effect of EWRs would be stronger for providers who participated in EWRs. As part of the follow-up survey we asked providers if they recalled participating in an EWR session (executive names were listed by the question). Response options included yes, no, and not sure. This resulted in three groups in the intervention group (EWRs-participant, EWRs-not sure if participant, and EWRs-not a participant) and three groups in the control group (control EWRs-participant, control EWRs-not sure if participant, and control EWRs-not a participant). Some providers in the control units received EWRs because they temporarily worked in an intervention unit on the day EWRs occurred. We hypothesized that mean safety climate scores would be higher for providers in the intervention EWRs-participant group than in the control EWRs-not a participant group.

#### Mean safety climate scores

Differences in mean SCS scores were tested using random intercept linear models to account for the non-independence of research participants nested within clinical units. These models were fit using SAS PROC MIXED. Model assumptions were verified by examining univariate histograms and residual skewness and kurtosis values, and by generation of residual-by-predicted value plots.

#### Analyses of group differences on individual items

Individual survey item responses consist of the ordered categorical values that represent the scale response labels of "disagree strongly", "disagree slightly", "neutral", "agree slightly", and "agree strongly". Intervals between these responses need not be equal. For this reason, we employed a cumulative odds logistic regression model to compare groups' odds of agreement on each survey question. The performance of the cumulative odds model benefits from ten or more responses per survey category [14]. Response categories were collapsed as needed to attain this objective (described in the note following Table 4). To obtain proper standard errors, confidence intervals, and *p*-values adjusted for clustering of respondents within clinical areas, we employed the SURVEYLOGISTIC procedure in SAS 9.1.3 to fit the cumulative odds models.

**Table 4:** Effect of executive walk rounds on nurses responses to survey items: Distributions of responses by survey item and EWRs participation.

**Survey Item**	**EWRs Participant n (%)**					**EWRs Non-participant n (%)**				
	**Disagree Strongly**	**Disagree Slightly**	**Neutral**	**Agree Slightly**	**Agree Strongly**	**Disagree Strongly**	**Disagree Slightly**	**Neutral**	**Agree Slightly**	**Agree Strongly**
1. The culture of this clinical area makes it easy to learn from the mistakes of others.	0	2 (2.44)	15 (18.29)	22 (26.83)	43 (52.44)	6 (3.13)	21 (10.94)	50 (26.04)	53 (27.60)	62 (32.29)
2. Medical errors are handled appropriately in this clinical area.	3 (3.61)	2 (2.41)	12 (14.46)	12 (14.46)	54 (65.06)	3 (1.54)	17 (8.72)	38 (19.49)	48 (24.62)	89 (45.64)
3. The senior leaders in my hospital listen to me and care about my concerns.	3 (3.57)	6 (7.14)	15 (17.86)	26 (30.95)	34 (40.48)	29 (14.65)	23 (11.62)	40 (20.20)	54 (27.27)	52 (26.26)
4. The physician and nurse leaders in my area listen to me and care about my concerns.	0	6 (7.14)	11 (13.10)	27 (32.14)	40 (47.62)	10 (5.15)	20 (10.31)	40 (20.62)	56 (28.87)	68 (35.05)
5. Leadership is driving us to be a safety-centered institution.	1 (1.19)	0	15 (17.86)	21 (25.00)	47 (55.95)	4 (2.03)	18 (9.14)	48 (24.37)	59 (29.95)	68 (34.52)
6. My suggestions about safety would be acted upon if I expressed them to management.	1 (1.19)	4 (4.76)	14 (16.67)	25 (29.76)	40 (47.62)	11 (5.58)	26 (13.20)	39 (19.80)	50 (25.38)	71 (36.04)
7. Management/Leadership does not knowingly compromise safety concerns for productivity.	3 (3.61)	5 (6.02)	14 (16.87)	24 (28.92)	37 (44.58)	17 (8.76)	21 (10.82)	33 (17.01)	53 (27.32)	70 (36.08)
8. I am encouraged by my colleagues to report any patient safety concerns I may have.	2 (2.41)	3 (3.61)	4 (4.82)	20 (24.10)	54 (65.06)	1 (0.51)	13 (6.63)	25 (12.76)	55 (28.06)	102 (52.04)
9. I know the proper channels to direct questions regarding patient safety.	0	1 (1.23)	4 (4.94)	19 (23.46)	57 (70.37)	0	2 (1.03)	17 (8.72)	60 (30.77)	116 (59.49)
10. I receive appropriate feedback about my performance.	2 (2.38)	4 (4.76)	10 (11.90)	26 (30.95)	42 (50.00)	6 (3.06)	24 (12.24)	38 (19.39)	59 (30.10)	69 (35.20)
11. I would feel safe being treated here as a patient.	0	2 (2.53)	13 (16.46)	21 (26.58)	43 (54.43)	5 (2.56)	20 (10.26)	35 (17.95)	62 (31.79)	73 (37.44)
12. Briefing personnel before the start of a shift (i.e., to plan for possible contingencies) is an important part of patient safety.	0	0	5 (6.10)	19 (23.17)	58 (70.73)	2 (1.04)	4 (2.07)	19 (9.84)	34 (17.62)	134 (69.43)
13. Briefings are common here.	1 (1.30)	3 (3.90)	15 (19.48)	24 (31.17)	34 (44.16)	13 (7.30)	13 (7.30)	40 (22.47)	45 (25.28)	67 (37.64)
14a. I am satisfied with availability of clinical leadership (physician).	1 (1.25)	5 (6.25)	9 (11.25)	21 (26.25)	44 (55.00)	9 (4.62)	26 (13.33)	32 (16.41)	55 (28.21)	73 (37.44)
14b. I am satisfied with availability of clinical leadership (nursing).	2 (2.44)	3 (3.66)	8 (9.76)	31 (37.80)	38 (46.34)	12 (6.25)	13 (6.77)	33 (17.19)	60 (31.25)	74 (38.54)
14c. I am satisfied with availability of clinical leadership (pharmacy).	5 (6.33)	7 (8.86)	18 (22.78)	21 (26.58)	28 (35.44)	20 (10.53)	32 (16.84)	42 (22.11)	51 (26.84)	45 (23.68)
15. This institution is doing more for patient safety now than it did one year ago.	0	1 (1.32)	11 (14.47)	22 (28.95)	42 (55.26)	11 (6.11)	6 (3.33)	59 (32.78)	59 (32.78)	45 (25.00)
16. I believe that most adverse events occur as a result of multiple system failures, and are not attributable to one individual's actions.	0	4 (5.13)	8 (10.26)	24 (30.77)	42 (53.85)	8 (4.17)	16 (8.33)	23 (11.98)	65 (33.85)	80 (41.67)
17. The personnel in this clinical area take responsibility for patient safety.	0	0	8 (9.76)	23 (28.05)	51 (62.20)	2 (1.04)	9 (4.66)	26 (13.47)	75 (38.86)	81 (41.97)
18. Personnel frequently disregard rules or guidelines that are established for this clinical area.	30 (37.50)	10 (12.50)	14 (17.50)	12 (15.00)	14 (17.50)	47 (23.98)	43 (21.94)	39 (19.90)	40 (20.41)	27 (13.78)
19. Patient safety is constantly reinforced as the priority in this clinical area.	0	1 (1.27)	9 (11.39)	16 (20.25)	53 (67.09)	4 (2.02)	14 (7.07)	37 (18.69)	59 (29.80)	84 (42.42)

## Results

A total of 1119 providers (67%) completed the baseline survey and 1,000 (55%) the post EWRs survey. There was no difference in safety climate between the EWR and control units when all providers were analyzed. We only report results for nurses (Licensed Vocational Nurses, Registered Nurses, and Nurse Managers) because we could not detect an effect of EWRs on safety climate scores for other providers. We randomized 23 units, 12 to the control group and 11 to the EWR group. Prior to EWRs, 547 nurses returned surveys; after EWRs, 598 nurses returned surveys. The types of nurses whose attitudes were measured post-EWR, their ages, and experience in the organization are shown in Table 2.

**Table 2:** Demographic characteristics of the post walk rounds nurse respondents.

	**Walk rounds n (%)**	**Control n (%)**
Total	260	338

**Type of Nurse**		
LVN	30 (11.5)	30 (8.9)
RN	207 (79.6)	291 (86.1)
Nurse Manager	23 (8.8)	17 (5.0)
**Age (years)**		
Less than 30	86 (33.1)	87 (25.7)
30–34	39 (15.0)	45 (13.3)
35–39	34 (13.1)	42 (12.4)
40–44	42 (16.1)	68 (20.8)
45 and over	54 (20.8)	89 (26.3)
Missing	5 (1.9)	7 (2.1)
**Years in this hospital**		
Less than 1	52 (20.0)	44 (13.0)
1–2	41 (15.8)	46 (13.6)
3–7	61 (23.5)	96 (28.4)
8–12	49 (18.8)	90 (26.6)
13–20	24 (9.2)	34 (10.1)
21 and over	17 (6.5)	10 (3.0)
Missing	16 (6.2)	18 (5.3)

Providers and executives discussed specific issues during the Walk rounds and 12 themes were identified (Table 3). The hospital addressed 8 of the 12 themes prior to the follow up administration of the SCS. Some actions to address these themes were limited to units from which the theme arose (n = 4), other actions affected all units (n = 5). The follow up survey was administered before the hospital addressed: 1) difficulties in transitioning patients from Emergency Department to ICUs; 2) problems with TPN orders in neonatal intensive care; 3) Inconsistent application of the falls prevention program; and 4) difficulties caring for medical patients with significant psychiatric problems. Providers were notified of these changes through staff meetings.

**Table 3:** Themes identified during walk rounds.*

1. Medication ordering policy not followed (handwriting illegible, cannot identify ordering MD, etc.)
2. The medicaton administration record is not always reconciled with the most recent orders
3. Active interventions not maintained on the electronic medical record
4. Need to improve discharge education for patients on anticoagulants
5. House officers need better supervision when conducting procedures and nurses need a way to identify house officer training level and which procedures are appropriate for that level.
6. Difficulties in caring for medical patients with significant psychiatric problems
7. Management of overweight patients (inadequate equipment, difficulty turning, transporting)
8. Problems with TPN orders in neonatal intensive care
9. Inconsistent application of the falls prevention program
10. Difficulties in transitioning patients from Emergency Department to intensive care units (timing of transfer, use of different intravenous drug concentrations)
11. Improper use of oxygen tanks when patients transported
12. Beds not well maintained (wheel locks malfunction, frayed electrical cords)

*The hospital had not responded to items 6, 8, 9, and 10 prior to the follow-up safety climate survey.

### Mean safety climate score comparisons

All 547 nurses measured pre-EWR returned useable safety climate data; by contrast, 570 participating nurses returned useable safety climate data following EWRs. Before EWRs the mean safety climate scores for nurses were similar in the control units and EWR units (78.97 and 76.78, P = 0.458) as were percent positive scores (64.6% positive and 61.1% positive). After EWRs the mean safety climate scores and percent positive scores were not significantly different in the control units and EWR units (77.93 and 78.33, P = 0.854) and (56.5% positive and 62.7% positive). However, nurses in the control group who did not participate in EWRs (n = 198) had lower safety climate scores than nurses in the intervention group who did participate in an EWR session (n = 85) (74.88 versus 81.01, P = 0.02; 52.5% positive versus 72.9% positive).

### Comparisons of individual safety climate scale items

The distributions of responses by survey item and by EWRs participation are shown in Table 4. Compared to nurses who did not participate, nurses in the Experimental group who reported participating in EWRs responded more favorably on the items hypothesized a priori to be most sensitive to EWRs (Table 5). All five items showed statistically significant differences in odds of agreement with items for the EWR-participant compared to Control-Non-participant groups: This institution is doing more for patient safety now, than it did one year ago (item 15, OR = 3.82, p < .001); 2) The senior leaders in my hospital listen to me and care about my concerns (item 3, OR = 2.15, p = .012); 3) Patient safety is constantly reinforced as the priority in this clinical area (item 19, OR = 2.79, p = .001); 4) Leadership is driving us to be a safety-centered institution (item 5, OR = 2.48, p = .002); and 5) I would feel safe being treated here as a patient (item 11, OR = 2.05, p = .002). Examination of the odds ratios listed in Table 5 shows that nurses in the EWRs-participant group exhibited more favorable evaluations of safety climate through their responses to the individual safety climate items than did nurses in the control EWRs-not a participant group on 14 out of 21 items (Table 5; note that the items are labeled 1–19, but item 14 has three parts.

**Table 5:** Effect of executive walk rounds on survey items for nurses: Odds of agreement with an item for EWR Participants compared to EWR non-participants.

**Survey Item**	**OR**	**95% CI**
1. The culture of this clinical area makes it easy to learn from the mistakes of others.	2.50	1.15 – 5.42
2. Medical errors are handled appropriately in this clinical area.	2.05	0.97 – 4.35
*3. The senior leaders in my hospital listen to me and care about my concerns.	**2.15**	**1.18 – 3.92**
4. The physician and nurse leaders in my area listen to me and care about my concerns.	1.89	1.13 – 3.16
*5. Leadership is driving us to be a safety-centered institution.	**2.48**	**1.39 – 4.45**
6. My suggestions about safety would be acted upon if I expressed them to management.	1.89	1.04 – 3.43
7. Management/Leadership does not knowingly compromise safety concerns for productivity.	1.56	0.90 – 2.70
8. I am encouraged by my colleagues to report any patient safety concerns I may have.	1.74	1.01 – 2.75
9. I know the proper channels to direct questions regarding patient safety.	1.62	0.87 – 3.03
10. I receive appropriate feedback about my performance.	1.98	1.23 – 3.20
*11. I would feel safe being treated here as a patient.	**2.05**	**1.31 – 3.19**
12. Briefing personnel before the start of a shift (i.e., to plan for possible contingencies) is an important part of patient safety.	1.16	0.65 – 2.07
13. Briefings are common here.	1.56	0.89 – 2.73
14a. I am satisfied with availability of clinical leadership (physician).	2.14	1.35 – 3.42
14b. I am satisfied with availability of clinical leadership (nursing).	1.62	0.90 – 2.93
14c. I am satisfied with availability of clinical leadership (pharmacy).	1.75	1.13 – 2.72
*15. This institution is doing more for patient safety now than it did one year ago.	**3.82**	**1.87 – 7.81**
16. I believe that most adverse events occur as a result of multiple system failures, and are not attributable to one individual's actions.	1.70	1.17 – 2.48
17. The personnel in this clinical area take responsibility for patient safety.	2.29	1.26 – 4.17
18. Personnel frequently disregard rules or guidelines that are established for this clinical area.	0.78	0.48 – 1.28
*19. Patient safety is constantly reinforced as the priority in this clinical area.	**2.79**	**1.50 – 5.21**

Notes:

1. N = 274 non-missing cases.

2. Odds ratios and 95% confidence intervals are derived via a five category cumulative odds logistic regression model fit using the SURVEYLOGISTIC procedure in SAS version 9.1.3. Confidence intervals are adjusted for clustering of participants within clinical areas. Each analysis is based on five ordered categories of response options: "strongly disagree", "disagree", "neither agree nor disagree", "agree", "strongly agree", with the following exceptions: Some items (1, 2, 5, 8, 10, 11, 15, 16, 17, 19) had fewer than ten respondents who endorsed "strongly disagree" or "disagree"; for these items the "strongly disagree" and "disagree" categories were pooled to yield n-per-category greater than or equal to ten cases. Similarly, items 9 and 12 required pooling of the "strongly disagree", "disagree", and "neither agree nor disagree" categories to yield ten or more cases per category.

3. The odds ratios indicate the odds of an EWR participant having more agreement with an item than a EWR non-participant

4. Item 18 is reversed scored.

## Discussion

To our knowledge, this is the first published study to show that EWRs can improve safety climate among some providers in hospitals. Nurses in the intervention group who participated in EWRs had higher safety climate scores than non-participants in the control group. However, EWRs conducted in this manner did not result in higher safety climate scores for *all *nurses in the intervention units compared to the control units, suggesting a limited spill-over effect from nurses who participated in EWRs to those who did not participate. Furthermore, we did not detect an effect of EWRs on the attitudes of other provider types. This may be due to lack of power to detect a difference (there were relatively small numbers of some other provider types) or because EWRs or the safety climate survey may be less relevant to non-nursing providers.

### Implications

Safety culture has been identified as a key safety practice for healthcare by the National Quality Forum and other experts because poor safety culture may contribute to unsafe practices. We found that EWRs can positively influence safety climate (one component of safety culture) among nurses who participated in EWRs. Our study should be encouraging and informative to hospitals and researchers who are experimenting with the use of EWRs.

The effect of EWRs on safety climate of participating nurses was detected after a short intervention (3 months) and after relatively minor changes in care processes. Future research should address the "dose-response" relationship between EWRs and safety climate. EWRs may need to be conducted more frequently, address a broader range of topics, address a larger audience during rounds, or occur for a longer duration than in our study (once a month for three months) to influence other providers and to have an impact on overall unit safety climate. A key goal should be to expose as many providers as possible (both day and night shifts) to the EWRs because the effect on attitudes may not diffuse throughout a clinical unit (although we had limited power to detect this effect). The effect of EWRs may also be modified by the actions taken to correct or address safety problems raised by providers during the EWRs. The actions taken after this set of EWRs were relatively minor in scope suggesting that the EWRs themselves may be important mediators of safety attitudes. More visible or more effective actions could result in greater improvements in safety climate, both among providers who participated in EWRs and those who did not.

### Limitations

Our ability to detect differences between the control and EWR units was limited by sample size at the clinical unit level (23 units in total) and because some nurses in control units participated in EWRs because they were floating in another unit while a EWR occurred. Sample sizes of non-nursing groups also limited our ability to detect changes after EWRs.

Providers were not randomized so our provider level analysis that found a positive effect of EWRs may be biased. There may be factors that influenced participation in EWRs that also caused or were associated with more positive attitudes. The main determinant of participation was whether or not the nurse worked days or nights (EWRs were only done during the day). Day-shift nurses may respond differently than night-shift nurses to EWRs. Other reasons for non-participation for day-shift nurses were that EWRs occurred on days that they did not work, the nurse could have been too busy with patient care, or they could have chosen not to participate. The EWR schedule was determined by the executives, not nurse managers.

Generalizability is limited because we report findings from only one urban tertiary care hospital and because the EWR effect may be mediated by the individual executives of this hospital. Executives were advised about the purpose of EWRs and given questions to help guide their discussion (Table 1) but there was variability in the details of each EWR. This makes our findings more generalizable in that it mimics what most hospitals will do (use multiple executives), but also less generalizable in that others cannot exactly replicate our intervention. Future research should investigate executive effects to better establish what executives do well during EWRs, what level of executive is most effective, and whether a particular background (e.g., clinical/nonclinical) makes a difference. Randomized trials usually assume that the intervention (like a drug) is stable over time, and that the effect of the intervention is independent of other factors in the environment (although there are notable exceptions [15]). EWRs is a social intervention that varies depending upon the individual(s) conducting EWRs and the culture of the units receiving the intervention. Therefore, a formative evaluation that reports details of the interactions among executive characteristics (leadership style, gender, position in organization) and the units (size, recent safety issues, work routines) would be informative, as would a unit level analysis of safety climate scores. Future studies should also consider comparing EWRs to visits by executives who simply introduce themselves and ask how things are going. This may better isolate the effect of EWRs over and above the effect due to an executive appearing on the unit to talk.

Due to practical considerations for this particular hospital, we did not include physicians. Based upon our experience with other EWR efforts, inclusion of physicians in EWRs may lead to greater improvements in the unit-level safety climate because physicians may be positively influenced by the EWRs, and because they may be leaders in units so a positive attitude change could affect others on the unit.

Many readers will wonder if improved safety climate leads to reductions in errors and adverse events. We did not collect such data but experience at other institutions suggests that as error rates and lengths of stay decline because of interventions, safety climate scores increase [16]. Others may see safety climate attitudes as important outcomes regardless of their relationships with errors and adverse events, and research in aviation suggests a relationship among attitudes and performance [12,13].

## Conclusion

EWRs have a positive effect on the safety climate attitudes of nurses who participate in the walk rounds sessions. EWRs may need to be performed more frequently or for a longer period of time than in this study in order to have a broader influence on provider attitudes. Future research should also look in detail at the interactions among executive and unit characteristics to better understand this safety intervention. By embracing the knowledge of front line providers, engaging them directly in improvement efforts, and aligning the concerns of providers and leaders [17,18], EWRs may improve safety climate and the broader construct of safety culture.

## Competing interests

The author(s) declare that they have no competing interests.

## Pre-publication history

The pre-publication history for this paper can be accessed here:


