# Correction: The effect of executive walk rounds on nurse safety climate attitudes: A randomized trial of clinical units [ISRCTN85147255]

**DOI:** 10.1186/1472-6963-5-46

**Published:** 2005-06-10

**Authors:** Eric J Thomas, J Bryan Sexton, Torsten B Neilands, Allan Frankel, Robert L Helmreich

**Affiliations:** 1 Department of Internal Medicine, The University of Texas Medical School at Houston, Houston, TX, USA; 2 Department of Anesthesiology and Critical Care Medicine, Johns Hopkins Quality and Safety Research Group, The Johns Hopkins University School of Medicine, Baltimore, USA; 3 Center for AIDS Prevention Studies (CAPS), University of California, San Francisco, San Francisco, CA, USA; 4 Partners Healthcare System, Prudential Tower, Boston, MA, USA; 5 Department of Psychology, The University of Texas at Austin, Austin, TX, USA

## 

Following publication of this article [1], the study was included in the International Standard Randomised Controlled Trial Number (ISRCTN) Register and assigned the number ISRCTN85147255.

## Pre-publication history

The pre-publication history for this paper can be accessed here:

http://www.biomedcentral.com/1472-6963/5/46/prepub
